# Erratum: Hearing the Shape of the Ising Model with a Programmable Superconducting-Flux Annealer

**DOI:** 10.1038/srep40651

**Published:** 2017-01-31

**Authors:** Walter Vinci, Klas Markström, Sergio Boixo, Aidan Roy, Federico M. Spedalieri, Paul A. Warburton, Simone Severini

Scientific Reports 4: Article number: 5703; 10.1038/srep05703 published online: 07 16 2014; updated: 01 31 2017.

This article was originally published under a CC BY-NC-SA 4.0 license, but has now been made available under a CC BY 4.0 license. The PDF and HTML versions of the paper have been modified accordingly.

